# Hybrid Pareto artificial bee colony algorithm for multi-objective single machine group scheduling problem with sequence-dependent setup times and learning effects

**DOI:** 10.1186/s40064-016-3265-3

**Published:** 2016-09-17

**Authors:** Lei Yue, Zailin Guan, Ullah Saif, Fei Zhang, Hao Wang

**Affiliations:** 1 State Key Lab of Digital Manufacturing Equipment and Technology, HUST-SANY Joint Lab of Advanced Manufacturing, Huazhong University of Science and Technology, Wuhan, 430074 People’s Republic of China; 2 Department of Industrial Engineering, University of Engineering and Technology, Taxila, Pakistan

**Keywords:** Group scheduling, Multi-objectives, Hybrid Pareto artificial bee colony algorithm, Sequence dependent setup, Learning effect, Taguchi method

## Abstract

Group scheduling is significant for efficient and cost effective production system. However, there exist setup times between the groups, which require to decrease it by sequencing groups in an efficient way. Current research is focused on a sequence dependent group scheduling problem with an aim to minimize the makespan in addition to minimize the total weighted tardiness simultaneously. In most of the production scheduling problems, the processing time of jobs is assumed as fixed. However, the actual processing time of jobs may be reduced due to “learning effect”. The integration of sequence dependent group scheduling problem with learning effects has been rarely considered in literature. Therefore, current research considers a single machine group scheduling problem with sequence dependent setup times and learning effects simultaneously. A novel hybrid Pareto artificial bee colony algorithm (HPABC) with some steps of genetic algorithm is proposed for current problem to get Pareto solutions. Furthermore, five different sizes of test problems (small, small medium, medium, large medium, large) are tested using proposed HPABC. Taguchi method is used to tune the effective parameters of the proposed HPABC for each problem category. The performance of HPABC is compared with three famous multi objective optimization algorithms, improved strength Pareto evolutionary algorithm (SPEA2), non-dominated sorting genetic algorithm II (NSGAII) and particle swarm optimization algorithm (PSO). Results indicate that HPABC outperforms SPEA2, NSGAII and PSO and gives better Pareto optimal solutions in terms of diversity and quality for almost all the instances of the different sizes of problems.

## Background

Group technology (GT) is a well-known method used to improve the production efficiency in manufacturing and engineering management through exploiting similarities of different products and exploiting similar activities in their designs and production processes. GT was first proposed by Mitrofanov (1966) and Opitz (1970) and later many manufacturing companies have taken advantage of GT to enhance productivity (Webster and Baker 1995; Logendran et al. 2005; Keshavarz and Salmasi 2014). Variety of scheduling models used GT in which set of similar jobs are divided into subsets, called families or groups. Each job in a group contains similar technological requirements in terms of tooling and setups. This can eliminate the time of setups between the jobs in a single group and increase the production efficiency. Jobs grouping advantage has increased the research in group scheduling (GS) and has attracted numerous researchers due to their significant application in industries. Different GS research problems in manufacturing environment have been addressed in literature. For example, single-machine GS problem (SMGS) (Webster and Baker 1995; Kuo and Yang 2006; Kuo 2012; Wu et al. 2008), GS in flowshop environment (FSGS) (Logendran et al. 2005; Gelogullari and Logendran 2010; Solimanpur and Elmi 2011; Costa et al. 2014) etc. More recent works on GS problems in different manufacturing environment have also been presented in literature (Keshavarz et al. 2015; Neufeld et al. 2015; Ji et al. 2016; Egilmez et al. 2016; Adressi et al. 2016).

In many industries, there exists frequent changeover of jobs on machines which needs setup time. If the frequent change of jobs occurs on the bottleneck resources of the production system, it can cause a large amount of waste of time. According to the theory of constraints (TOC), the performance of complex manufacturing systems often depends mostly on the bottleneck machines of the production system. Therefore, scheduling with setup times on the bottleneck machines plays a critical role for the enterprise because it is primarily cause for delays in the delivery of customer orders. Production schedules of the system often rely on management of these setup times on bottleneck resources. The setup time includes sequence-independent setup times and sequence-dependent setup (SDS) times. Setup time is sequence-independent if its duration depends only on the current job to be processed. Setup time is sequence-dependent if setup time depends on both the current and the immediately preceding job. The presence of SDS has increased the complexity of industrial scheduling problem. Group technology has the advantage that, no machine setups are needed between two consecutively scheduled jobs in the same group due to similarities in operations. However, setup time is required between processing of jobs from different groups which is called as group setup. In most real-world problems, the group setup time is considered as sequence dependent. Therefore, SDS has been investigated in literature for GS problems to enhance the advantage of GT. The sequencing of groups in an order that the two consecutive groups in the sequence can require less changes in the machines setup. This can reduce the SDS time between different groups in the group scheduling. Group scheduling with SDS has been studied by limited researchers. In recent literature, Costa et al. (2014), Neufeld et al. (2015) and Salmasi et al. (2011) considered a group scheduling problem with sequence dependent setup times to minimize the makespan. Keshavarz et al. (2015) investigated a flexible flowshop sequence-dependent group scheduling problem with an objective to minimize total completion time. Moreover, a sequence dependent group scheduling problem on unrelated-parallel machines with a combined objective of makespan and total weighted tardiness has also been addressed (Bozorgirad and Logendran 2012). Khamseh et al. (2015) presented a model which integrates group scheduling problem with sequence-dependent setups and preventive maintenance activities in order to minimize the total completion time. Zandieh and Karimi (2011) presented a multi-objective group scheduling problem with SDS times by minimizing total weighted tardiness and the maximum completion time simultaneously. Due to significant application of group scheduling, current research investigates the problem of group scheduling with SDS on single machine environment.

In classical scheduling models, the processing time of jobs is assumed as fixed and the schedule is made on the fixed processing time of jobs. However, in many realistic situations where manual workers perform operations, due to repetition of production operations, the actual processing time of jobs can be reduced as compared to its initial value due to “learning effect”. When the new workers are assigned to process some jobs, the worker can take different time as compared to the time they take after several times repetition of the process of the same job on machines. Learning effect can cause change in the processing time of jobs with repetition and the schedule which is based on fixed value of the processing time of jobs might be optimal for the fixed processing time value. The change in processing time can cause different waiting time of jobs in the schedule and can give different value of the performance objectives as compared to predetermined schedule. Scheduling with learning effect is significant and therefore, it has received considerable attention in recent years (Kuo and Yang 2006; Zhu et al. 2011; Huang et al. 2011; Wang et al. 2008; Yin et al. 2009; Li et al. 2013). Due to the significance application of learning effect, it has also been studied by several researchers in group scheduling problems (Kuo and Yang 2006; Yang and Yang 2010; Kuo 2012; Bai et al. 2012; Zhu et al. 2011; Yang 2011). However, they have not considered the SDS in the group scheduling. In literature, some studies have considered group scheduling with SDS but have not involved learning effect in their models (Janiak et al. 2005; Schaller 2001; Salmasi et al. 2011; Keshavarz and Salmasi 2014; Keshavarz et al. 2015; Neufeld et al. 2015). A limited research work found in literature has considered both learning effects and SDS in group scheduling problem simultaneously. Low and Lin (2012) considered a single machine group scheduling problem with past sequence dependent setup (PSDS) and learning effect. They considered makespan and the total completion time as objectives in their studies. The PSDS they considered is significant and more suitable for the cases where the setup time of the newly insert job depends on all the previous jobs that are scheduled to process before it. The past sequence dependent setup time is more applicable in the PC Board industries (Koulamas and Kyparisis 2008; Wang 2008; Low and Lin 2012). However, in most other industries, setup time depends only on the newly entered job in the sequence and the last scheduled job before this job in most of the production environment (Dudek et al. 1974). For example, in the manufacturing industries of heavy machinery, SDS is more significant where the SDS exists on production machines and depends only on the two consecutive jobs of the sequence irrespective of the other jobs in the schedule. For example, SDS exists in the jobs on different machines in SANY Heavy Industry Company in Changsha, China and Yu Tong BUS Company in Zhengzhou, China which are producing heavy construction machinery and buses and other transportation machinery respectively. The SDS times occurs in these companies and is considered in the current research.

In literature studies on group scheduling problems, most of the research optimized either single objective or linear combination of more than one objective with giving certain weight to each objective (Neufeld et al. 2015; Costa et al. 2014; Salmasi et al. 2011; Keshavarz et al. 2015; Karimi et al. 2011). However, in most of the companies, more than one objective is desired to optimize and in most of the cases, the desired objectives are conflicting and companies require to optimize these conflicting objectives simultaneously. Simultaneous consideration of more than one conflicting objective is significant in most of these companies. Therefore, current research used two conflicting objectives including makespan and total weighted tardiness to optimize simultaneously for the current research single machine group scheduling problem with SDS times and LE for the heavy machinery manufacturing company environment.

Group scheduling problem with SDS is NP hard (Webster and Baker 1995; Janiak et al. 2005). In literature, different methods have been proposed to investigate group scheduling (Logendran et al.^2005^; Solimanpur and Elmi 2011; Adressi et al. 2016; Zhu et al. 2011) and group scheduling with SDS (Costa et al.^2014^; Keshavarz et al. 2015; Neufeld et al. 2015; Ji et al. 2016; Karimi et al. 2011; Salmasi and Logendran 2008; Sabouni and Logendran 2013; Anghinolfi and Paolucci 2009). For example, heuristics (Neufeld et al. 2015; Salmasi and Logendran 2008; Li et al. 2013), branch-and-bound procedure (Schaller 2001; Sabouni and Logendran 2013; Keshavarz et al. 2015), tabu search (Bozorgirad and Logendran 2012), particle swarm optimization (Anghinolfi and Paolucci 2009), imperialist competitive algorithm (Karimi et al. 2011), genetic algorithm (Zandieh and Karimi 2011; Adressi et al. 2016), etc. Recently, Karaboga (2005), proposed an artificial bee colony algorithm (ABC) which is a popular algorithm and it is based on the foraging behavior of honey bee swarm. ABC algorithm needs less control parameters, can be used for different kind of continuous and discrete problems and easy to implement. These features make it feasible and applicable in different areas of optimization problems. Therefore, it has been applied to permutation flowshop (Tasgetiren et al. 2011), flexible job shop (Li et al. 2011), large scale engineering optimization problems (Akay and Karaboga 2012), and constraint optimization problem (Ajorlou and Shams 2013) etc. In recent years there is little research work that has been done for multi objective optimization problems (Omkar et al. 2011; Akbari et al. 2012; Pan et al. 2011) etc. Zhang et al. (2013) proposed a hybrid ABC for flowshop problem and more recently Saif et al. (2014) proposed Pareto based ABC for multi objective optimization of simple assembly line balancing problem. However, their presented algorithm described above fits more for the type of problem in their study, which motivates us to introduce hybrid Pareto ABC (HPABC) algorithm for the current problem.

Current research is novel to consider group scheduling problem on a single machine with SDS consideration and learning effect. Moreover, multiple conflicting objectives including makespan and TWT are considered simultaneously to optimize and the Pareto optimal results are obtained. Furthermore, the considered problem in the current study has not, to date, been presented and solved using some recent meta-heuristics such as ABC algorithm. Moreover, the proposed HPABC algorithm is novel to solve the current research problem. The proposed HPABC algorithm employs some steps of genetic algorithm and incorporates the Pareto optimality in the original ABC algorithm for this problem and is novel.

Reset of the paper is organized as follows: “Problem description and formulation” section illustrates the problem formulation. “Hybrid Pareto artificial bee colony algorithm” section deals with the proposed HPABC algorithm. “Taguchi experimental design” section presents the data generation and test case specifications of the current problem, and then describes tuning of the parameters of the proposed algorithm using Taguchi method. “Experimental results” section illustrates computational experiments and results over five different categories of problems and makes results comparisons among three different algorithms by performance of some evaluation indexes. Finally, “Conclusions” section concludes the paper and presents some future aspects of the research.

## Problem description and formulation

**Fig. 1:**
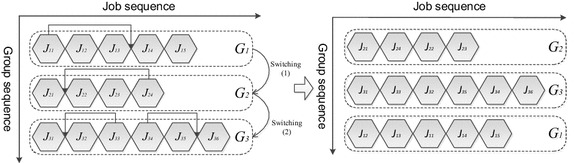
An abridged general view of group scheduling problem with SDS times

The group scheduling problem for a single machine with sequence dependent setup and learning effect can be formulated as follows. There are *n* jobs in *m* groups to be processed. Different numbers of jobs are grouped into families accordingly to the GT principles. Each group *G*_*i*_, for 1 ≤ *i* ≤ *m*, consists of a set of *n*_*i*_ jobs $\left\{ {J_{i1} ,J_{i2} , \ldots ,J_{{in_{i} }} } \right\}$. Assuming that all the jobs are available for processing at time zero on a continuously available machine. An abridged general view of group scheduling problem with SDS times which schedule the groups and the jobs in each groups simultaneously is indicated in Fig. 1 as follow.

The problem is developed using the following notations. Additional notations will be introduced when needed throughout the paper.

**Table:** .

Notations and abbreviations	
*m*	The number of groups
*n*_*i*_	The number of jobs in group *G*_*i*_
*n*	The total number of jobs, $\sum\nolimits_{i = 1}^{m} {n_{i} = n}$
*i*	Index used to represent a group
*h*	Index used to represent a group
*j*	Index used to represent a job
*r*	The job position in a group
*k*	The group position in a sequence
*r*_*i*_	The setup time if a job in group *i* is first scheduled in the sequence
*α*	Learning effect factor for jobs within a group, *α* > 1
*β*	Learning effect factor for jobs among groups, 0 < *β* ≤ 1
*GS*_*ih*_	The group setup time from group *i* to group *h*
*J*_*ij*_	The job *j* in group *i*, *j* = (1, 2, …, *n*_*i*_)
*P*_*ij*_	The normal processing time of a job *J*_*ij*_
*P*_*i*[*r*]_	The normal processing time of a job *J*_*i*[*r*]_ which is scheduled in the *r*th position in a sequence in group *G*_*i*_
$P_{ij}^{k,r}$	The actual processing time of a job *J*_*ij*_ is scheduled in the *r*th and in the *k*th group in a sequence
*d*_*ij*_	The due date of a job *J*_*ij*_
*w*_*ij*_	The weight of a job *J*_*ij*_ regarding the objective function
*C*_*ij*_	The completion time of job *J*_*ij*_
$C_{ij}^{k,r}$	The completion time of job *J*_*ij*_ which is scheduled in the *k*th group position and *r*th job position in a schedule
*C*_*max*_	The makespan of an instance
$T_{{J_{ij} }}$	The tardiness of job *J*_*ij*_, $T_{{J_{ij} }} = max\left\{ {0,C_{ij} - d_{ij} } \right\}$
*TWT*	The total weighted tardiness of all jobs of all groups
$$X_{h}^{k} \left\{ {\begin{array}{*{20}c} 1 \\ 0 \\ \end{array} } \right.$$	*If group h is processed at the kth position in the schedule* *Otherwise*
$$Y_{hj}^{l} \left\{ {\begin{array}{*{20}c} 1 \\ 0 \\ \end{array} } \right.$$	*If job j in group h is processed at lth position* *Otherwise*
$$X_{ijq} \left\{ {\begin{array}{*{20}c} 1 \\ 0 \\ \end{array} } \right.$$	*If job q is processed after job j in group i* *Otherwise*
$$X_{ih} \left\{ {\begin{array}{*{20}c} 1 \\ 0 \\ \end{array} } \right.$$	*If group h is processed after group i* *Otherwise*

*P*_*ij*_ is used to indicate the normal processing time of job *j* in group *i*. *r* and *k* denote the job position in the group and the group position in the group sequence respectively. In addition, *P*_*i*[*r*]_ represent the normal processing time of a job if it is scheduled in the *r*th position in the group *i* in the sequence. Both time-dependent and position-based learning effects are used to determine the actual processing time of a job in a specific job group (Low and Lin 2012). The actual processing time of a job in each group is a function of the sum of the normal processing times of the jobs already scheduled and the position of the corresponding group in the schedule. The actual processing time of a job *J*_*ij*_ that is scheduled in the *r*th position and in the *k*th group in a schedule, $P_{ij}^{k,r}$, is computed from Eq. (1).

1 $$P_{ij}^{k,r} = P_{ij} \left( {1 - \frac{{\sum\nolimits_{l = 1}^{r - 1} {P_{i\left[ l \right]} } }}{{\sum\nolimits_{l = 1}^{{n_{i} }} {P_{il} } }}} \right)^{a}\quad \beta^{k - 1} = P_{ij} \left( {\frac{{\sum\nolimits_{l = r}^{{n_{i} }} {P_{i\left[ l \right]} } }}{{\sum\nolimits_{l = 1}^{{n_{i} }} {P_{il} } }}} \right)^{a} \quad \beta^{k - 1} ,\quad \forall j,r = 1,2, \ldots ,n_{i} ,\quad \forall i,k = 1,2, \ldots ,m$$

Minimizing makespan is the first objective that we consider for this problem and is as:

2 $$Z_{1} = \hbox{min} \left( {\hbox{max} \left\{ {C_{ij} } \right\}} \right)$$

The second objective is to minimize total weighted tardiness as below:

3 $$Z_{2} = \hbox{min} \left( {TWT} \right)$$

where,

$$TWT = \sum\limits_{i = 1}^{m} {\sum\limits_{j = 1}^{{n_{i} }} {w_{ij} T_{{J_{ij} }} } }$$

Every group is located in only one position in the group schedule and all the groups must be included in the group schedule.

4 $$\sum\limits_{h = 1}^{m} {X_{h}^{k} = 1,\quad \forall h,k = 1,2, \ldots ,m}$$

5 $$\sum\limits_{k = 1}^{m} {\sum\limits_{h = 1}^{m} {X_{h}^{k} = m,\quad \forall h,k = 1,2, \ldots ,m} }$$

Each job in a group should be assigned only one position in jobs schedule in its group and all the jobs in the group should be sequenced in the schedule.

6 $$\sum\limits_{j = 1}^{{n_{h} }} {Y_{hj}^{l} = 1,\quad \forall j,l = 1,2, \ldots ,n_{h} \quad \forall h = 1,2, \ldots m}$$

7 $$\sum\limits_{l = 1}^{{n_{h} }} {\sum\limits_{j = 1}^{{n_{h} }} {Y_{hj}^{l} = n_{h} ,\quad \forall j,l = 1,2, \ldots ,n_{h} \quad \forall h = 1,2, \ldots m} }$$

For m number of groups to schedule, there occurs total of (m − 1) number of sequence dependent setups.

8 $$\sum\limits_{h = 1}^{m} {\sum\limits_{i = 1}^{m} {X_{ih} = m - 1,\quad \forall h,i = 1,2, \ldots ,m,\quad i \ne h} }$$

Completion time of a job *J*_*iq*_ located in the first position among jobs in group *i* and the group *i* is located in first position in the group schedule.

9 $$C_{iq}^{1,1} = \sum\limits_{i = 1}^{m} {X_{i}^{1} } \left( {r_{i} + \sum\limits_{q = 1}^{{n_{i} }} {Y_{iq}^{1} P_{iq} } } \right),\quad \forall i = 1,2, \ldots ,m\quad \forall q = 1,2, \ldots ,n_{i}$$

Completion time of a job *J*_*is*_ located in any position among jobs in group *i* and the group *i* is located in any position in the group schedule.

10 $$C_{is}^{1,r} = C_{it}^{1,r - 1} + \sum\limits_{i = 1}^{m} {\sum\limits_{s = 1}^{{n_{i} }} {Y_{is}^{r} X_{i}^{1} P_{is} \left( {\frac{{\sum\nolimits_{l = r}^{{n_{i} }} {P_{i\left[ l \right]} } }}{{\sum\nolimits_{l = 1}^{{n_{i} }} {P_{il} } }}} \right)^{a} } } ,\quad \forall i = 1,2, \ldots ,m\quad \forall s,t,r = 1,2, \ldots ,n_{i} ,\quad s \ne t$$

Completion time of a job *J*_*hq*_ located in first position among jobs in group *h* and the group *h* is located in second position in the group schedule.

11 $$C_{hq}^{2,1} = C_{is}^{{1,n_{i} }} + \sum\limits_{i = 1}^{m} {\sum\limits_{h = 1}^{m} {X_{i}^{1} X_{h}^{2} X_{ih} GS_{ih} + \sum\limits_{h = 1}^{m} {\sum\limits_{q = 1}^{{n_{h2} }} {Y_{hq}^{1} X_{h}^{2} P_{hq} \beta } \quad \forall i,h = 1,2, \ldots ,m,i \ne h\quad \forall q = 1,2, \ldots ,n_{h} \quad \forall s = 1,2, \ldots ,m} } }$$

Completion time of a job *J*_*hs*_ located in any position among jobs in group *h* and the group *h* is located in second position in the group schedule.

12 $$C_{hs}^{2,r} = C_{ht}^{2,r - 1} + \sum\limits_{h = 1}^{m} {\sum\limits_{s = 1}^{{n_{h} }} {Y_{hs}^{r} X_{h}^{2} \left( {\frac{{\sum\nolimits_{l = r}^{{n_{h} }} {P_{h\left[ l \right]} } }}{{\sum\nolimits_{l = 1}^{{n_{h} }} {P_{hl} } }}} \right)^{a} } } \beta ,\quad \forall h = 1,2, \ldots ,m\quad \forall s,t,r = 1,2, \ldots ,n_{h} ,\quad s \ne t,\;r \ne 1$$

Completion time of a job *J*_*gq*_ located in first position among jobs in group *g* and the group *g* is located in any position in the group schedule.

13 $$C_{gq}^{k,1} = C_{of}^{{k - 1,n_{o} }} + \sum\limits_{g = 1}^{m} {\sum\limits_{o = 1}^{m} {X_{g}^{k} X_{o}^{k - 1} X_{og} GS_{og} + \sum\limits_{g = 1}^{m} {\sum\limits_{q = 1}^{{n_{g} }} {Y_{gq}^{1} X_{g}^{k} P_{gq} \beta^{k - 1} } } } } ,\quad \forall g,o,k = 1,2, \ldots ,{\text{m}},\;g \ne o\quad \forall q = 1,2, \ldots ,n_{g} \quad \forall f = 1,2, \ldots ,n_{o}$$

Completion time of a job *J*_*gs*_ located in any position among jobs in group *g* and the group *g* is located in any position in the group schedule.

14 $$C_{gs}^{k,r} = C_{gt}^{k,r - 1} + \sum\limits_{g = 1}^{m} {\sum\limits_{s = 1}^{{n_{g} }} {Y_{gs}^{r} X_{g}^{k} P_{gs} \left( {\frac{{\sum\nolimits_{l = r}^{{n_{g} }} {P_{g\left[ l \right]} } }}{{\sum\nolimits_{l = 1}^{{n_{g} }} {P_{gl} } }}} \right)^{a} } } \beta^{k - 1} ,\quad \forall g,k = 1,2, \ldots ,m\quad \forall s,t,r = 1,2, \ldots ,n_{g} ,\quad s \ne t,\;r \ne 1$$

The objectives of minimizing makespan and minimizing total weighted tardiness are illustrated in Eqs. (2) and (3) respectively. The constraints of the proposed problem are shown in Eqs. (4)–(8). Completion time of any job *J*_*gs*_ from group *g* in a given schedule is given from Eqs. (9) to (14) described above.

## Hybrid Pareto artificial bee colony algorithm

Artificial bee colony algorithm (ABC), proposed by Karaboga (2005), is a popular algorithm and it is based on the foraging behavior of honey bee swarm. ABC algorithm is composed of three kinds of bees called, employee bee, onlooker bees and scout bees. The number of employee bees and onlooker bees are equal. The food source in ABC algorithm represents a solution of the problem and the nectar amount of the food source indicates the corresponding fitness of the solution. In ABC algorithm employee bees travels in the field and taste different food sources and takes their nectar amounts. The nectar amount of the food sources identifies the value of the objectives or nectar value of the food sources. Employee bees informs this nectar value to the onlooker bees which are waiting in the dance area in the hive. Onlooker bee investigates the employee bees and selects the best food source from them. They also decide the future direction of the employee bee to travel for further search of the food sources. The employee bee which gets the same value of nectar amount from the food sources it searches for known number of cycles (called limit cycles), is turned to a scout bee and scout bee find the new direction of travel to search food sources randomly. This cycle is repeated for known number of algorithm cycles and the best food source ever found is considered as near optimal solution of the considered optimization problem.

**Fig. 2:**
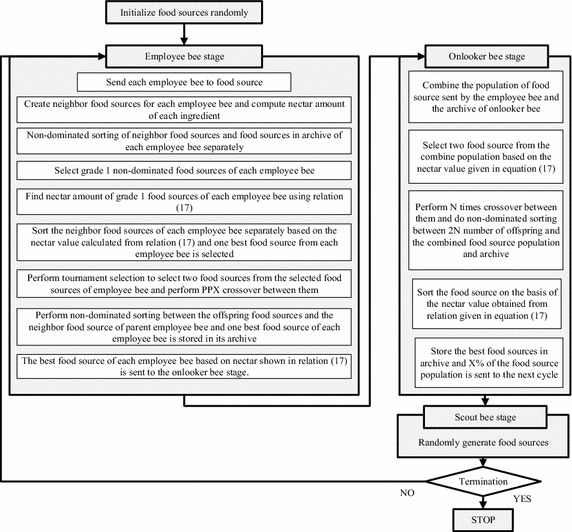
Flowchart of the proposed HPABC algorithm

The problems investigated in literature are quite different from the current research problem of simultaneous group scheduling and job sequencing problem. The solution of current problem is desired to have sequence of different group of jobs and in each group the jobs sequence is also needed. The solution requirement of the current optimization problem is different and therefore a new food source representation is needed to study group scheduling and job sequencing in each group simultaneously. The flowchart of the proposed HPABC is shown in Fig. 2 and the step wise procedure of the proposed HPABC algorithm is presented in this section.

### Encoding of food source

**Fig. 3:**
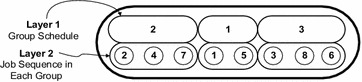
Food source representation of the group scheduling and job sequencing problem

The food source in the current problem is designed to consider both the sequence of groups and schedule of jobs in each group. The food source for the current problem is composed of two layers. The first layer of food source represents the permutation encoding of the group of jobs and is called as layer 1 of the food source. The second layer of the food source represents the sequence of jobs in each group and is called as layer 2 of food source as shown in Fig. 3. It can be seen from Fig. 2 that there are three groups of jobs in the food source of layer 1. The food source presented in Fig. 2 indicates that second group of jobs can process at the first priority and later the group 1 and at the end group 3 will processing. The layer 2 of the corresponding food source indicates that the sequence of jobs in first group (i.e. group 2) is 2, 4 and 7, sequence of jobs in group 1 is 1, 5 while the sequence of jobs in group 3 is 3, 8, and 6 respectively. The proposed encoding of food source in the current group scheduling problem is significant to make several schedules of groups and in each group, different sequences of the jobs are also formed and this kind food sources can be tasted by the employee bees to identify the best food source in the proposed HPABC algorithm.

### Initializing food sources

Food source population is generated randomly to include different kind of food sources for tasting. These food sources are tested and employee bee tastes these food sources if the solution represented in the food source satisfies all the constraints of the problem. Otherwise, the food source is again created randomly. The number of food source generated is equal to the number of employee bees.

### Send employee bees

**Fig. 4:**
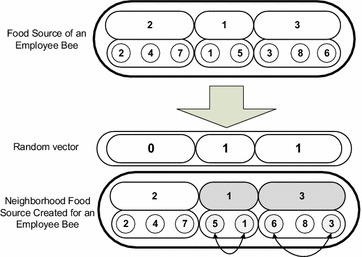
Creation of a neighborhood food source of an employee bee

**Fig. 5:**
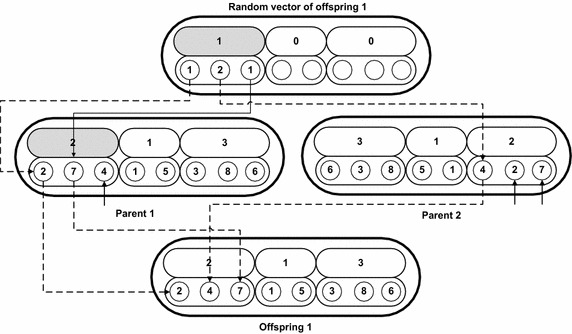
PPX crossover in layer *2* to create offspring *1*

The employee bee phase of proposed HPABC is composed of following steps:

*Step 1*: Send each employee bee to its respective food source to taste it and get the nectar amount.
*Step 2*: In the proposed HPABC algorithm, each employee bee creates and taste known number of neighbor food sources of the original food source given to it. The neighbors of food source have same solution in their layer 1 while have different solutions in their layer 2. This can increase the possibility that for each schedule of groups, different job sequences can be formed. In order to create neighbor food sources, a random vector is generated in which the number of elements is equal to the number of groups and the numbers appearing in each element of this vector has value of 0 or 1. The 0 value corresponds to the condition that while making a neighbor food source, the sequence of jobs in the corresponding group is not changed. Whereas the value of 1 in an element of the random vector corresponds to the condition that the jobs of the corresponding group can change their sequence to make a neighbor. Swap mutation is used to change the position of jobs in the groups which are allowed to change their job positions according to the values (i.e. 0 or 1) appearing in the random vector. The random vector and the procedure of swap mutation to change the sequence of jobs for different groups for a food source to create its neighborhood food a source is indicated in Fig. 4. It can be seen from Fig. 4 that the random vector has elements equal to the number of groups in the food source i.e. 3 elements. The elements in the random vector which have value of 1 allowed their corresponding groups to change the corresponding jobs sequences in them. As can be seen from Fig. 4 that the second and third element of random vector has values of 1 and the corresponding groups in the food source are job group 1 and group 3 and they are appeared in grey color. The jobs appearing in these groups in the layer 2 of the food source can change the position of jobs in them by swap mutation, i.e. job 5 and job 1 are interchanged in the group 1 and job 6 and job 3 are interchanged in group 3.
  Each employee bee creates *E*_*neb*_ number of its neighbors and for each neighbor, there is a new random vector. Current problem is multi objective optimization problem and therefore, each food source is required to be observed on all objectives. Therefore, nectar amount of food source ingredients is computed in this stage, each ingredient corresponds to an objective. The nectar amount of food source ingredients are illustrated in Eqs. (15) and (16).
  15 $$Nec_{1} = C_{\hbox{max} }$$
  16 $$Nec_{2} = \sum\limits_{i = 1}^{m} {\sum\limits_{j = 1}^{{n_{i} }} {w_{ij} T_{{J_{ij} }} } }$$
  where, $T_{{J_{ij} }} = \hbox{max} \left\{ {0,C_{ij} - d_{ij} } \right\}$, *d*_*ij*_ is the due date of job *J*_*ij*_, *w*_*ij*_ is the weight related to the job *J*_*ij*_.
*Step 3*: In this step, non-dominated sorting of the food source neighbors of each employee bee along with the stored food source of each employee bee (if there is some food source in archive of each employee bee) is performed separately (Deb et al. 2002). In non-dominated sorting, a food source *S* dominates another food source *F* i.e. *S* ≺ *F* if food source *S* is better than the food source *F* in all of its ingredients. Further, *S* is strictly better than *F* in at least one of the food source ingredient value. Non-dominated solutions from the food source neighbors of each employee bee are separately identified from the population of neighborhood food sources of each employee bee. These non-dominated food sources of each employee bee are separately graded. The grade 1 non-dominated food sources are those to which no other solutions can dominate. The grade 1 food non-dominated food sources might be more than one for each employee bee.
*Step 4*: For each employee bee there is possibility that they can have more than one food source as non-dominated in grade 1 and in this situation, a food source which is in the middle range of the Pareto front is given priority in the proposed HPABC algorithm because the middle values on the Pareto front are more near to the optimal values. Furthermore, there is requirement of the diversity in the solutions and therefore, a new value of the nectar value is designed here which can combine the effect of the middle point food sources on the Pareto front and the diversified food sources from the front. Equation (17) indicates the nectar amount of the food source *i*.
  17 $$Nec_{i} = \left( {W_{cd} \times C_{d} } \right)_{i} + \left( {W_{mc} \times MS} \right)_{i}$$
  where, *W*_*cd*_ is the weightage given to the crowding distance of the food sources, *C*_*d*_ is the crowding distance (Deb et al. 2002) of the non-dominated food sources, *W*_*mc*_ is the weightage given to the Pareto points on the middle of the Pareto front and *MS*_*i*_ is the middle score which can be computed from the relation shown in Eq. (18).
  18 $$MS_{i}^{e} = d_{i,1} \times d_{1,2} ,\quad \forall 1 < i < h$$
  where, *h* is the number of Pareto points on the front for the Pareto solutions of the neighbors of an employee bee, $MS_{i}^{e}$ is the selection function of a Pareto point *i*. The larger value of $MS_{i}^{e}$ can give a solution which is more in the middle on the Pareto front. $MS_{i}^{e}$ defines the product of the Euclidean distance of a Pareto point *i* on the front from the two extreme points on the Pareto front. Neighbor food sources of each employee bee are sorted on the basis of the value of the nectar amount computed from Eq. (17) and one best food source from the neighbors of each employee bee is selected.
*Step 5*: From the population of the selected food source of each employee bee (population has one best food source from each employee bee), tournament selection is performed to select two food sources from them and they are named as parent food sources. Precedence preservative crossover (PPX) operation is performed between them to share information between them. The PPX operation is performed on the layer 2 of the parent food sources of the employee bees. In order to perform PPX crossover, a random vector is formed similar in structure with the food sources. The elements in the layer 1 of this random vector have values of 0 or 1 and each element corresponds to a job group. For example, the first element of this random vector corresponds to the group appearing on first position in layer 1 of the food source. The value of 0 in the element of first layer shows that the corresponding group has no crossover between the parent food sources. If there appears 1 in the element of the layer 1 of this random vector, means there is crossover operation in the corresponding job group of the parent food sources and the crossover is performed for the group appearing in parent 1 food source. The layer 2 of the random vector indicates the values of either 1 or 2. The value 1 corresponds to parent 1 and 2 corresponds to the second parent. If there appears 1 in the element of the layer 2 of the random vector, it indicates that the corresponding value of the element in the offspring 1 is filled with the value appearing in the parent 1 and same value is deleted from the parent 2, as shown in Fig. 5. In order to generate the second offspring, the element values of the random vector in the layer 2 are reversed, i.e. replace 1 with 2 and replace 2 with 1 to make a new random vector to create random vector for the second offspring. The proposed PPX crossover operation is indicated in Fig. 5. It can be seen from Fig. 5 that there are three groups in parent 1 and parent 2 of the food sources. The random vector has three elements in layer 1 and the values appearing in it are 1, 0 and 0. The 1 value indicates that there is crossover operation in the group which is appearing at the first location in layer 1 of the parent food source 1 i.e. group 2. Therefore, the crossover is performed between the parent food source for the group which is appeared at position 1 in the parent 1 food source and the same group in parent 2 i.e. group 2. The random vector is reversed to replace 1 with 0 and replace 0 with 1 for the creation of second offspring. The layer 1 of random vector for second offspring indicates the groups of parent 2 which can have crossover operation.
*Step 6*: In this step, non-dominated sorting is performed between the neighbor food sources and offspring food sources of the two selected employee bee (the employee bee from which parent food sources are obtained) separately and the one best non-dominated food source on the basis of nectar value shown in Eq. (17) from each is stored in their archive. Each employee bee has a separate archive to store the selected neighbor of each employee bee separately. The selected food source from the non-dominated sorting of each employee bee neighbor food sources, they all are stored in an archive of each employee bee and their archive is updated after each cycle of the algorithm.
*Step 7*: One best food source neighbor from each employee bee which has maximum nectar value of Eq. (17) is selected and the selected neighbor food source from each employee bee is sent to the onlooker bees.

### Send onlooker bee

**Fig. 6:**
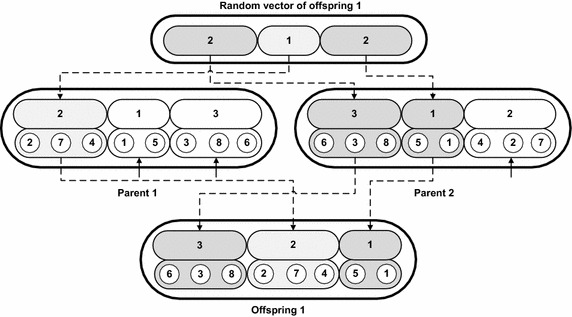
PPX crossover in layer *1* to create offspring food sources

Onlooker bee phase of the proposed HPABC algorithm is composed of the following steps:

*Step 1*: Onlooker bee stage of the proposed HPABC have a separate archive to store the best food sources found after finishing the onlooker bee stage. In this step, the food sources in the archive and the food sources given by the employee bee are combined to make a single population and non-dominated sorting is performed between them.
*Step 2*: The food sources which are appearing on the middle of the Pareto front are given priority and two of the best food sources from the Pareto front are obtained based on the nectar value appearing in Eq. (17).
*Step 3*: The selected two food sources are considered as parent food sources in onlooker bee phase. They are allowed to crossover for N times to create 2N number of offspring. The crossover is allowed to be performed only in layer 1 of the food sources. The procedure of crossover in layer 1 is indicated in Fig. 6. It can be seen from Fig. 6 that a random vector is created which can give the values of either 1 or 2. These values correspond to parent 1 and parent 2 respectively. When there appears value of 1 in the random vector, the corresponding element of the offspring is filled with the same elements in layer 1 and layer 2 of the parent food source 1 and similar group is deleted from the food source of parent 2. For example, the first element of the random vector is 2, it means the first element of the offspring will be filled with the first element of the parent 2 and the first element with layer 1 and layer 2 of parent 2 is copied to the first element of the offspring 1 and it is deleted from the parent 1 (as described by a small arrow in element containing the same group of jobs from parent 1 food source, i.e. group 3 is deleted from parent 1 once it is appeared in the offspring 1). The same procedure is followed to create the second offspring but the random vector is reversed, i.e. the value 1 appearing in the random vector is changed to 2 and the value appearing as 2 in random vector is changed to 1 for making a new random vector for offspring 2.
*Step 4*: Non-dominated sorting is performed between 2 N number of offspring, the food sources in the onlooker bee stage and the food sources in archive to get a Pareto front. The nectar value of the food sources is computed using relation given in Eq. (17) and the food sources are sorted on the basis of this nectar amount.
*Step 5*: The best food sources are stored in the archive for next cycle of the algorithm and the X % of the population of the food sources for the employee bee for next cycle of algorithm is taken from this archive and remaining is obtained from scout bee.

### Send scout bee

The scout bees are used to introduce diversity in the food source population and they introduce new food sources to the employee bees. Scout bee can create random food sources and give this information to the employee bees.

## Taguchi experimental design

Artificial bee colony algorithm, like most other searching algorithms, is mainly influenced by values of parameters. These parameters can be set manually or by applying different setting approaches such as full factorial experiment. This is a comprehensive approach but it would lose its efficiency by increasing the number of parameters (Montgomery 2000; Karimi et al. 2011), while in Taguchi method, a large number of decision variables would be tuned through a small number of experiments.

Taguchi method is used to design set of experiments in the form of an orthogonal array (OA). In OA, different levels of each parameter are defined and for each experiment there exist different combination of parameter levels to make different set of experiments. Each experiment has different levels of parameters consisting of different values. The number of columns in this matrix represents different parameters and the rows represents the number of experiments, each containing different set of parameters. These set of experiments with each containing different of levels of parameters, signal to noise (S/N) ratio is determined. S/N is the ratio of the objective function value obtained for an experiment with the variance value of the objective function. Taguchi method is used to determine best set of levels of all parameters of algorithm which can give maximum value of the S/N, i.e. best objective function value with less variations in its values. This method can identify the robust values of parameters which can be used for different instances of the problems.

### Data generation and test case specifications

The proposed HPABC algorithm is tested against several test problems. These test problems are much closer to the real-world problems. The main purpose of applying group scheduling techniques in production is to decompose the complex production problems. Thus, in industrial environment neither too many groups nor too many jobs in each group are expected to be assigned. According to relevant previous research, the maximum number of groups consider in current study is set equal to 20, and the maximum number of jobs in each group is set equal to 16. The number of groups is varied from 2 to 4, 5 to 8, 9 to 12, 13 to 16, and 17 to 20, for small, small medium, medium, large medium, and large problem instances, while the number of jobs in each group is a random integer taken from a discrete uniform (DU) distribution, DU [2, 4], DU [5, 7], DU [8, 10], DU [11, 13], and DU [14, 16] for small, small medium, medium, large medium, and large problems, respectively. The experiments are implemented on these five sizes of problem: small, small medium, medium, large medium, large which are shown in the Table 1. The specifications of required data for all the problems are as follows:

**Table 1:** Characteristics of different size of test problem

Size of problems	Factor	
	Number of groups (*m*)	Number of jobs in a group (*n*)
Small	2–4	U [2,4]
Small medium	5–8	U [5,7]
Medium	9–12	U [8,10]
Large medium	13–16	U [11,13]
Large	17–20	U [14,16]

- Processing times of jobs are made from DU [5, 25]
- Setup times between groups are generated from DU [5, 50]
- Defining proper due dates can positively affect the performance of the algorithms on the basis of previous work (Bozorgirad and Logendran 2012; Zandieh and Karimi 2011;). Two different factors are introduced to define due dates: tardiness factor (*τ*), and due date range factor (*R*). The tardiness factor (*τ*) is used to create loose or tight due dates, and *τ* is defined as $1 - \bar{d}/C_{max}$, where $\bar{d}$ is the average due date and *C*_*max*_ is the maximum completion time of all jobs. Tight or loose due dates can be obtained by large or small value of *τ* respectively. Moreover, the due date range factor (*R*) decides the variability of due dates. The range factor (*R*) is equal to (*d*_*max*_ − *d*_*min*_)/*C*_*max*_, where *d*_*min*_ is the minimum due date among all the jobs, and *d*_*max*_ is the maximum one. Different combinations of *τ* and *R* can provide different characteristics for randomly generated due dates. In current research, the values of *τ* and *R* are set to 0.4 and 0.6 severally which can provide small medium and wide range due dates. Then the due dates are uniformly distributed over the interval $[ {\bar{d} - R\bar{d}, \bar{d}}]$ with probability *τ* and over the interval $[ {\bar{d},\bar{d} + \left( {C_{max} - \bar{d}} \right)R} ]$ with probability (1 − *τ*).
- Job weights are generated from uniform integer distribution [1,4]
- The learning effect indexes are set as *α* = 1.5 and *β* = 0.9

### Tuning of proposed algorithm parameters with Taguchi method

To begin with the tuning of parameters, the parameters which can affect the performance of the results of proposed HPABC are identified. These factors include, the size of population of the food source, the number of neighborhoods of the algorithm and the maximum number of algorithm cycles. These three parameters are named here as population size, neighborhoods and cycles respectively. The parameter values are set against different levels which are illustrated in Table 2. It can be seen from Table 2 that each column of the table indicates different values of the parameters and their corresponding levels. For example, level 2 of the first parameters represents that the number of food sources in the experiment is 60 while level 2 of the second parameter indicates that number of neighborhoods of the algorithm is 30 and level 2 of the third parameter indicates the number of cycles of the algorithm, i.e. 100. The number of experiments for three parameters with each containing 5 levels runs for 10 times for each instance. Total 4750 (19 × 25 × 10) runs of the proposed HPABC algorithm are carried out to obtained the best level of parameters for different size of problems.

**Table 2:** Effective parameters of the proposed algorithm

Levels	Population size	Number of neighborhoods	Maximum number of algorithm cycles
1	40	20	50
2	60	30	100
3	80	40	150
4	100	50	200
5	120	60	250

**Table 3:** Orthogonal array (OA) for Taguchi design of experiments for the proposed algorithm

Experiment	Level of parameters		
	Population size	Number of neighborhoods	Maximum number of algorithm cycles
1	1	1	1
2	1	2	2
3	1	3	3
4	1	4	4
5	1	5	5
6	2	1	2
7	2	2	3
8	2	3	4
9	2	4	5
10	2	5	1
11	3	1	3
12	3	2	4
13	3	3	1
14	3	4	5
15	3	5	2
16	4	1	4
17	4	2	5
18	4	3	1
19	4	4	2
20	4	5	3
21	5	1	5
22	5	2	1
23	5	3	2
24	5	4	3
25	5	5	4

**Fig. 7:**
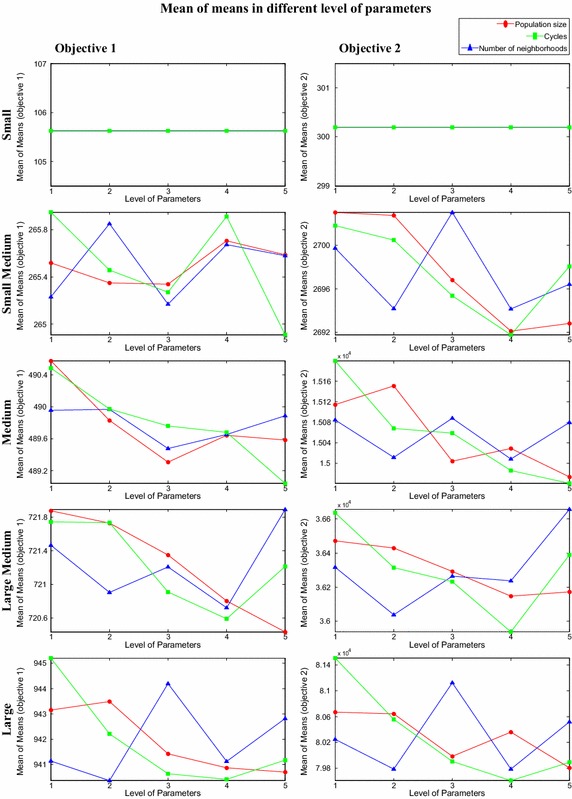
Mean value of means for different level of parameters

**Fig. 8:**
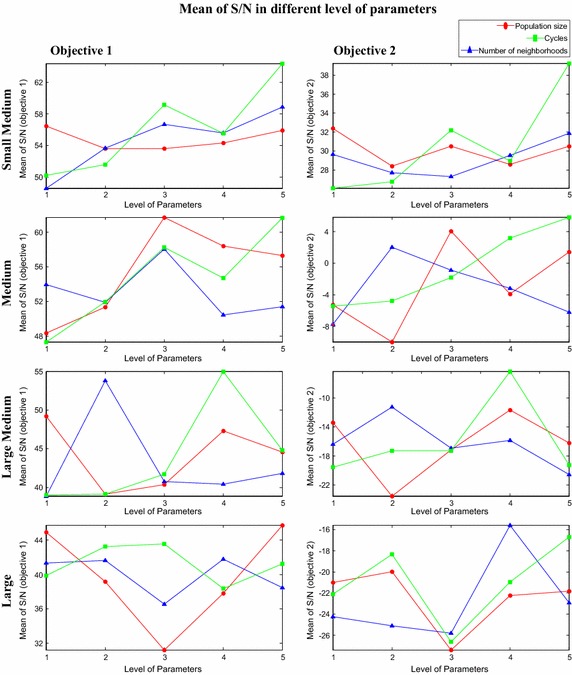
Mean value of S/N for different level of parameters

In the current experiment design, each problem is tested according to different level of parameters as mentioned in the proposed OA, as shown in Table 3 and the corresponding values of the two objective functions are computed. Once each problem is tested according set of parameters as given in OA, the mean value of the objectives, for each level of each parameter is computed for each problem. For example, the mean value of objectives for the parameter ‘population size’ at level 1 is obtained from first five experiments of the OA matrix. Similarly, mean value for parameter ‘population size’ at level 2 is acquired by taking the average of objective values obtained from the next five experiments. Similar procedure is employed to get mean value of objectives against each parameter for each level. Then mean of mean objective values (called mean of means) for each level of each category of problems is computed. Furthermore, the measured values that are obtained through the experiments are transformed into signal-to-noise (S/N) ratio. Actually this ratio is the amount of variation in the response variable. Signal-to-noise ratio can be categorized in different sets according to its characteristics: continuous or discrete; nominal-is-best, smaller-the-better, or larger-the-better. Based on current scheduling problem features, the current research applies nominal-is-best. The considered S/N value is indicated in Eq. (19).

19 $$\left( {{S \mathord{\left/ {\vphantom {S N}} \right. \kern-0pt} N}} \right)_{\text{nominal}} = 10\log \left( {\frac{{(mean)^{2} }}{{\left( {Variance} \right)^{2} }}} \right)$$

where, (mean)^2^ indicates the mean value of the optimizing objective and (variance)^2^ is the variance value in the optimizing objectives. S/N values for each objective of different problems are calculated according to OA and then mean value of S/N of each objective for each level of parameter is computed. Later, mean value of S/N values (called mean of S/N) for each level of each category of problems is computed. In the experiments, the mean S/N values of small size of problems are infinite due to Zero value of variance. However, mean value of means and mean value of S/N for considered problems are indicated in Figs. 7 and 8 respectively. Graphical method is employed here to identify the specific level of different parameters for each category of problems.

In the current case the level of parameter which gives small value of the optimizing objectives is preferred because objective functions are the minimizing objectives. Moreover, the level of parameter at which maximum value of S/N is obtained is preferred. The optimum level of parameter for each category of problem is obtained by observing both mean value of means and mean of S/N values of all objectives for each category of problem. The optimum level of parameters for each category of problem is illustrated in Table 4.

**Table 4:** Optimum level of parameters of proposed HPABC for each category of problem

Size of problems	Level of parameters		
	Population size	Number of neighborhoods	Maximum number of algorithm cycles
Small	1	1	1
Small medium	3	4	3
Medium	3	2	5
Large medium	5	2	4
Large	5	4	5

## Experimental results

In this section, performance of the proposed HPABC algorithm is tested using the optimum level of parameters obtained in the previous section. Several instances of different categories of problem which are presented before are analyzed using HPABC algorithm and other three famous multi-objective optimization algorithms in literature, i.e. non-dominated sorting genetic algorithm II (NSGAII) (Deb et al. 2002), the improved strength Pareto evolutionary algorithm (SPEA2) (Zitzler et al. 2001) and particle swarm optimization algorithm (PSO) (Kennedy and Eberhart 1995). The parameters of SPEA2, NSGAII and PSO used for different size of problems are also obtained from different runs of experiment and the parameters values which can give good results for SPEA2, NSGAII and PSO are selected for them. The selected values of parameters of the three considered algorithms for the tested instances are illustrated in Table 5. ‘Pop’ is used to represent population size, ‘Nei’ indicates Number of neighborhoods, and ‘Cyc’ is a shortened form of Maximum number of algorithm cycles. Each experiment of each instance is run 10 times by each algorithm. The results of the proposed HPABC algorithm are compared with that obtained from SPEA2, NSGAII and PSO. The proposed HPABC, SPEA2, NSGAII and PSO are all coded in Visual C# and run on an Intel Core i7, 3.4 GHz CPU, 4 GB RAM computer. The performance of proposed HPABC algorithm is compared with SPEA 2, NSGA II and PSO algorithm based on different metrics including diversity and quality of solutions, inverted generational difference and spacing of Pareto points on the Pareto fronts. The comparison of results based on each comparison metric is indicated in this section.

**Table 5:** Parameters for HPABC, SPEA2, NSGA II and PSO algorithm for different categories of problems

Size of problems	Parameters								
	HPABC			SPEA2		NSGAII		PSO	
	Pop	Nei	Cyc	Pop	Cyc	Pop	Cyc	Pop	Cyc
Small	40	20	50	60	50	60	100	60	50
Small medium	80	50	150	80	200	80	200	40	200
Medium	80	30	250	80	300	60	300	60	300
Large medium	120	30	200	100	300	100	250	80	250
Large	120	50	250	120	400	100	400	100	350

### The diversity and the quality of non-dominated solutions

In order to assess the performance of algorithms, the measures of diversity and quality which have been firstly applied by Hyun et al. (1998) are used in current study. The measures of diversity and quality are also used by Zandieh and Karimi (2011) called as, qualitative and quantitative measures. Both Hyun et al. (1998) and Zandieh and Karimi (2011) have presented the relation to determine the quality of Pareto. However, their studies have not considered common Pareto optimal solutions of the two different algorithms. There is possibility that true Pareto front can have some common optimal Pareto points both from HPABC and other comparison algorithms. Therefore, in current study a new measure of quality with a small improvement based on the previous work is presented. Since each algorithm finds out near Pareto optimal solutions, a solution found by algorithm A could dominate that found by another algorithm B, or vice versa. Putting together all the solutions found by A and B, non-dominated between them is performed. Some of Pareto points are the common solutions discovered by A and B simultaneously, some of them are only discovered by A or B respectively. Assuming that *N*_*A*_ and *N*_*B*_ are Pareto optimal solutions of algorithms A and B respectively, the combined Pareto front have *N*_*T*_ Pareto optimal solutions which is less than *N*_*A*_ + *N*_*B*_. *N*_*com*_ is defined as the number of common Pareto solutions found by A and B, *N*_*T*_^*A*^ and *N*_*T*_^*B*^ indicate the number of Pareto solutions of algorithms A and B in the combined Pareto front respectively. Diversity measure of each algorithm is its number of Pareto optimal solutions (*N*_*A*_ and *N*_*B*_ respectively) and is shown in Table 6. The quality measure (*Qua*_*i*_ ∀*i* = *A*, *B*) is a ratio calculated from the relation indicated in Eq. (20)

20 $$Qua_{i} = \frac{{N_{T}^{i} - N_{com} }}{{N_{T} - N_{com} }}\quad \forall i = A,B$$

**Table 6:** Results based on comparison of diversity

Problem	Size (*m* × *n*)	HPABC	SPEA2	NSGAII	PSO
Small	2 × [2–4]	9.00	6.40	9.00	9.00
	3 × [2–4]	3.00	1.60	3.00	3.00
	4 × [2–4]	3.00	1.90	3.00	3.00
Small medium	5 × [5–7]	22.60	8.20	14.70	15.30
	6 × [5–7]	28.90	13.20	18.50	17.20
	7 × [5–7]	31.50	14.10	16.40	14.80
	8 × [5–7]	12.60	6.40	8.80	9.10
Medium	9 × [8–10]	19.40	9.70	15.30	15.30
	10 × [8–10]	27.40	10.80	16.80	14.70
	11 × [8–10]	16.60	11.50	13.10	11.90
	12 × [8–10]	8.10	4.80	4.60	4.50
Large medium	13 × [11–13]	16.90	7.20	9.80	8.50
	14 × [11–13]	8.50	4.40	6.00	4.80
	15 × [11–13]	11.20	6.60	8.80	8.20
	16 × [11–13]	9.30	3.90	6.80	5.90
Large	17 × [14–16]	8.00	4.40	5.20	4.20
	18 × [14–16]	7.70	2.60	5.70	5.40
	19 × [14–16]	9.20	4.20	5.10	4.70
	20 × [14–16]	8.30	3.10	6.40	6.10
Avg.		13.64	6.56	9.21	8.72

The ratio may be used to indicate which algorithm is better in terms of solution quality. In this way, every pair of algorithms is compared, and the outcomes are shown in Table 7.

**Table 7:** Results based on comparison of quality

Problem	Size (*g* × *n*)	HPABC: SPEA2	HPABC: NSGAII	HPABC: PSO	NSGAII: SPEA2	NSGAII: PSO	PSO: SPEA2
Small	2 × [2–4]	(100.0:0.0)	Undefined	Undefined	(100.0:0.0)	Undefined	(100.0:0.0)
	3 × [2–4]	(100.0:0.0)	Undefined	Undefined	(100.0:0.0)	Undefined	(100.0:0.0)
	4 × [2–4]	(100.0:0.0)	Undefined	Undefined	(100.0:0.0)	Undefined	(100.0:0.0)
Small medium	5 × [5–7]	(91.8:8.2)	(61.1:38.9)	(73.5:26.5)	(78.2:21.8)	(68.2:31.8)	(72.5:27.5)
	6 × [5–7]	(71.1:28.9)	(59.5:40.5)	(63.7:36.3)	(57.4:42.6)	(53.5:46.5)	(55.1:44.9)
	7 × [5–7]	(90.2:9.8)	(77.6:22.4)	(82.5:17.5)	(60.2:39.8)	(56.9:43.1)	(59.2:40.8)
	8 × [5–7]	(86.4:13.6)	(58.0:42.0)	(53.6:46.4)	(59.2:40.8)	(43.5:56.5)	(59.0:41.0)
Medium	9 × [8–10]	(89.6:10.4)	(51.8:48.2)	(55.2:44.8)	(73.1:26.9)	(50.8:49.2)	(71.6:28.4)
	10 × [8–10]	(82.5:17.5)	(57.0:43.0)	(59.3:40.7)	(62.1:37.9)	(56.5:43.5)	(59.6:40.4)
	11 × [8–10]	(60.7:39.3)	(54.6:45.4)	(63.6:36.4)	(53.9:46.1)	(51.1:48.9)	(51.7:48.3)
	12 × [8–10]	(68.2:31.8)	(71.1:28.9)	(69.9:30.1)	(48.2:51.8)	(52.6:47.4)	(49.1:50.9)
Large medium	13 × [11–13]	(88.4:11.6)	(61.5:38.5)	(73.2:26.8)	(59.4:40.6)	(60.3:39.7)	(53.3:46.7)
	14 × [11–13]	(83.1:16.9)	(58.2:41.8)	(65.0:35.0)	(68.0:32.0)	(54.0:46.0)	(66.2:33.8)
	15 × [11–13]	(74.2:25.8)	(52.5:47.5)	(60.8:39.2)	(65.5:34.5)	(58.8:41.2)	(59.7:40.3)
	16 × [11–13]	(87.3:12.7)	(54.1:45.9)	(62.4:37.6)	(76.7:23.3)	(57.3:42.7)	(71.5:28.5)
Large	17 × [14–16]	(72.4:27.6)	(58.4:41.6)	(58.9:41.1)	(60.9:39.1)	(49.2:50.8)	(64.9:35.1)
	18 × [14–16]	(90.5:9.5)	(53.7:46.3)	(59.5:40.5)	(77.4:22.6)	(57.8:42.2)	(69.6:30.4)
	19 × [14–16]	(88.0:12.0)	(63.3:36.7)	(68.0:32.0)	(58.1:41.9)	(52.5:47.5)	(53.7:46.3)
	20 × [14–16]	(92.1:7.9)	(59.4:40.6)	(61.7:38.3)	(74.8:25.2)	(51.4:48.6)	(57.5:42.5)
Avg.		(85.08:14.92)	(59.49:40.51)	(64.42:35.58)	(70.16:29.84)	(54.65:45.35)	(67.06:32.94)

#### Comparison of diversity

The results based on comparison of diversity for the proposed HPABC, SPEA 2, NSGA II and PSO algorithms are indicated in Table 6. It can be seen from the Table 6 that in most of the studied problems, HPABC gives better results and gives more number of Pareto points as compared to NSGA II, SPEA 2 and PSO algorithm. For instance, for small medium size of problems, 31.50 average number of Pareto solutions are found for the case problem containing 7 groups by HPABC. However, SPEA 2 gives 14.10, NSGA II gives 16.40 and PSO gives 14.80 average number of Pareto solutions for the same problem instance. Moreover, for medium size of problems, 27.40 average number of Pareto solutions are obtained for the problem containing 10 groups instance by HPABC. However, SPEA 2 gives 10.80, NSGA II gives 16.80 and PSO gives 14.70 average number of Pareto solutions for the same problem respectively. Furthermore, for large medium size of problems, 16.90 average number of Pareto solutions are found for the case with 13 groups by HPABC while, SPEA 2 gives 7.70, NSGA II gives 9.80 and PSO gives 8.50 average number of Pareto solutions respectively for the same problem. In addition, for large size of problems, 9.20 average number of Pareto solutions are gained for the case with 19 groups by HPABC. However, SPEA 2 gives 4.20, NSGA II gives 5.10 and PSO gives 4.70 average number of Pareto solutions for large problem case with 19 groups respectively. Results shown in Table 6 indicates that, for small number of group problems, HPABC, NSGA II and PSO gives almost same average number of Pareto solutions. However, for rest of all problems belonging from each group size, HPABC outperforms NSGA II, SPEA 2 and PSO on the basis of diversity comparison results.

**Fig. 9:**
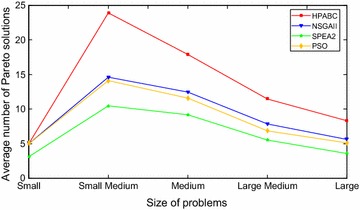
The average number of Pareto solutions for all instances of different size of problems obtained from the four different algorithms

The results based on comparison of diversity for different size of problems are indicated in Fig. 9. The average number of Pareto solutions for each size of problems by each algorithm is calculated from mean value of Pareto solutions of all instance of each size of problems and presented in Fig. 9 for each size category of problems. It can be seen from Fig. 9 that the number of Pareto points obtained from HPABC are larger than that from SPEA2, PSO and NSGAII against all size of problems and when the job groups become larger from small size to small medium size, the average Pareto solutions is increasing because of the extension of solution space. However, the average number of Pareto solutions is decreasing gradually from small medium size to large size due to the increasing complexity of the problem. These results indicate that, for small size problems, the Pareto solution points are less due to less search space of solutions for small problems. The number of Pareto solutions increases as the size of problem increases in the medium size problems and number of Pareto solutions decreases as the problem size increases and becomes larger than the average size problems. This is due to increase in complexity of problem as its size increases. This patterns of number of Pareto solutions is similar for HPABC, NSGA II, PSO and SPEA2. However, in all problem sizes, the number of Pareto solutions obtained from HPABC are more as compared to NSGA II, PSO and SPEA 2 algorithm.

#### Comparison of quality

The Pareto results obtained from HPABC, SPEA 2, PSO and NSGA II are also compared by computing the ratio of number of Pareto solutions obtained from one algorithm to the number of Pareto solutions obtained from other comparison algorithms. The results based on comparison of quality are indicated in Table 7. It can be seen from Table 7 that the ratios of quality for small size problems between HPABC and SPEA2 are (100:0 %) because HPABC obtained the true Pareto solutions for the small size of problems. Moreover, NSGAII and PSO also can find the true Pareto solutions for all the small size problems. Thus, the ratios of quality between NSGAII and SPEA2 and between PSO and SPEA2 are also (100:0 %). While the ratios between HPABC and NSGAII, HPABC and PSO and NSGAII and PSO are undefined on account of 0/0. However, for the rest of the problems HPABC can outperform NSGAII, PSO and SPEA2. For all instances in different size of problems, the average quality ratio between HPABC and SPEA2 is (85.08:14.92 %), and the average quality ratio is (59.49:40.51 %) between HPABC and NSGAII, while the average quality ratio is (64.42:35.58 %) between HPABC and PSO.

Overall results indicate that, HPABC can give the best performance both in diversity and in quality. NSGAII is demonstrated to be the second best both in terms of the number of non-dominated solutions and the quality of solutions, While PSO is tested to be the third best of the four algorithms. SPEA2 shows the worst results for both of the measures. However, for 12 groups instance of the medium size problem, the result of SPEA2 is a little better than NSGAII.

### Inverted generational distance

The current problem has two objectives, so the results of all instances of each category of problems from HPABC, SPEA2, PSO and NSGAII algorithms are sets of Pareto fronts. The inverted generational distance (GD) value is used to investigate the performance of the proposed HPABC, SPEA2, PSO and NSGAII by estimating the distance of elements in the Pareto optimal solutions from the true Pareto front. The value of GD is computed from the relation indicated in Eq. (21).

21 $$GD = \frac{{\sqrt {\sum\nolimits_{i = 1}^{h} {d_{i}^{2} } } }}{h}$$

where, *d*_*i*_ is the Euclidean distance between a Pareto optimal solution in the Pareto front and the nearest Pareto point in the true Pareto front, *h* is the number of Pareto optimal solutions in the Pareto front. The smaller value of GD indicates that the Pareto optimal solution is closer towards the true Pareto front and can give the near optimal solution.

**Fig. 10:**
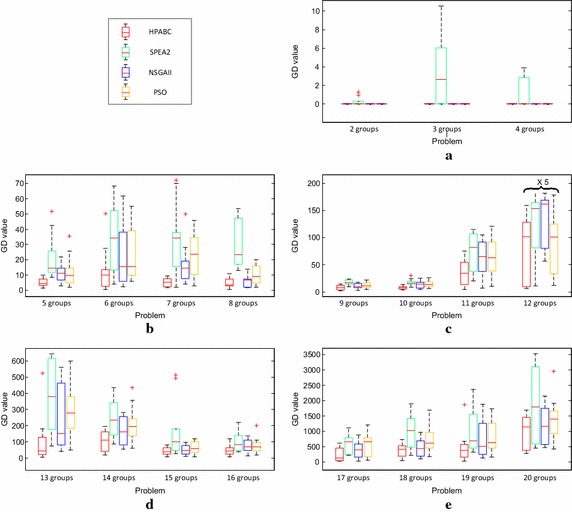
GD values of proposed HPABC, SPEA2, NSGAII and PSO algorithm for the problems from different size of problems. **a** GD values of 10 runs of each problem in Small size by the four algorithms respectively. **b** GD values of 10 runs of each problem in Small Medium size by the four algorithms respectively. **c** GD values of 10 runs of each problem in Medium size by the four algorithms respectively. **d** GD values of 10 runs of each problem in Large Medium size by the four algorithms respectively. **e** GD values of 10 runs of each problem in Large size by the four algorithms respectively

The comparison of HPABC, SPEA2, PSO and NSGAII on the basis of GD value for different size of problems at 10 runs of each problem is indicated in Fig. 10 using box plots. It can be seen from Fig. 10a that, the GD values of small size problems from 10 runs of HPABC, PSO and NSGAII respectively is always zero. These results indicate that the true Pareto fronts is found for the problems of small size by proposed HPABC, PSO and NSGAII. However, the GD values of SPEA2 for small size problems indicate the performance of SPEA2 is worse than HPABC, PSO and NSGAII. The GD results shown in Fig. 10b demonstrate that the proposed HPABC performs better than NSGAII, PSO and SPEA2 for small medium size problems. The error point of 6 groups case of HPABC indicate that a weak solution is found from 10 runs of this problem with HPABC. However, variations of GD values of HPABC is obviously less as compared to NSGAII, PSO and SPEA2. The GD values for medium size problems against HPABC, NSGA II, PSO and SPEA2 algorithm are shown in Fig. 10c. In Fig. 10c the problem containing 12 groups has large variations in the GD values for all comparison algorithms and GD values are divided by 5 to show in Fig. 10c. It can be seen from Fig. 10c that HPABC outperforms SPEA2, PSO and NSGAII in GD value for the medium size of problems. In addition, the GD values of these four algorithms are increasing with the increase of job groups respectively. The results based on GD value of different algorithms for large medium size problems and large size problems are indicated in Fig. 10d, e respectively. These two figures also show that HPABC can give the optimal solutions due to the smaller GD values for large medium size problems and large size problems.

### Spacing metric

Spacing metric is used to measure the distribution of Pareto points on the Pareto front. It is assumed that there are number of Pareto solutions on a front. Then SP can be computed from Eq. (22).

22 $$SP = \sqrt {\frac{1}{k - 1}\sum\limits_{u = 1}^{k} {\left( {d_{avg} - d_{u} } \right)^{2} } }$$

where, $d_{u} = \mathop {\hbox{min} }\nolimits_{v} \left[ {\mathop \sum \nolimits_{a = 1}^{O} \left| {Z_{a}^{u} - Z_{a}^{v} } \right|} \right],\quad \forall u,v = 1,2, \ldots ,k$, *k* indicates the number of solutions in the Pareto front, *d*_*avg*_ is the mean of all *d*_*u*_, *Z*_*a*_^*u*^ represents the value of objective *a*, *O* is the total number of objectives.

**Fig. 11:**
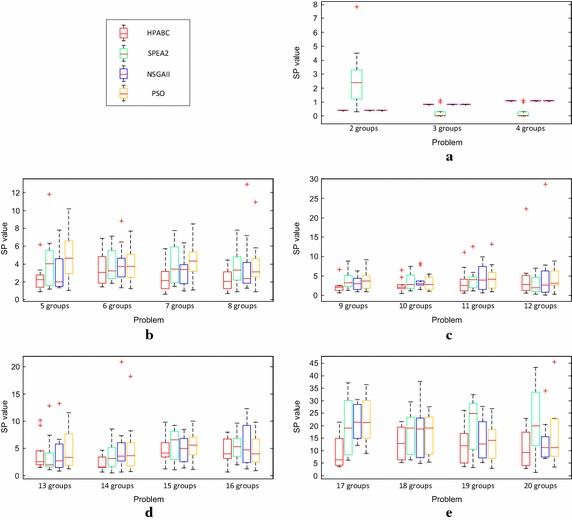
SP values of proposed HPABC, SPEA2, NSGAII and PSO algorithm for the problems from different size of problems. **a** SP values of 10 runs of each problem in Small size by the four algorithms respectively. **b** SP values of 10 runs of each problem in Small Medium size by the four algorithms respectively. **c** SP values of 10 runs of each problem in Medium size by the four algorithms respectively. **d** SP values of 10 runs of each problem in Large Medium size by the four algorithms respectively. **e** SP values of 10 runs of each problem in Large size by the four algorithms respectively

It can be seen from Eq. (22) that smaller value of the SP is desirable. Moreover, the zero value of SP indicates that all the Pareto points on the front are equidistant to each other and the Pareto points are evenly distributed on the front. The comparison of the performance of HPABC, SPEA 2, PSO and NSGAII algorithm based on the SP values from different 10 runs of experiment of different size of problems is indicated in Fig. 11 using box plots. It can be seen from Fig. 11a that, for small size problems, the performances of HPABC, PSO and NSGAII on the basis of SP values are same due to the same solution points found by these three algorithms. While the SP values of SPEA2 are smaller than HPABC, PSO and NSGAII for 3 groups instance and 4 groups instance. Nevertheless, it does not indicate that SPEA2 performs better than the other three algorithms because maybe only 1 or 2 solutions are found by SPEA2 at most runs of the problems. As shown in the rest of the figures in Fig. 11, for most of the problems in different size, the proposed HPABC gives better results of SP value as compared to SPEA2, PSO and NSGAII.

### Pareto fronts

**Fig. 12:**
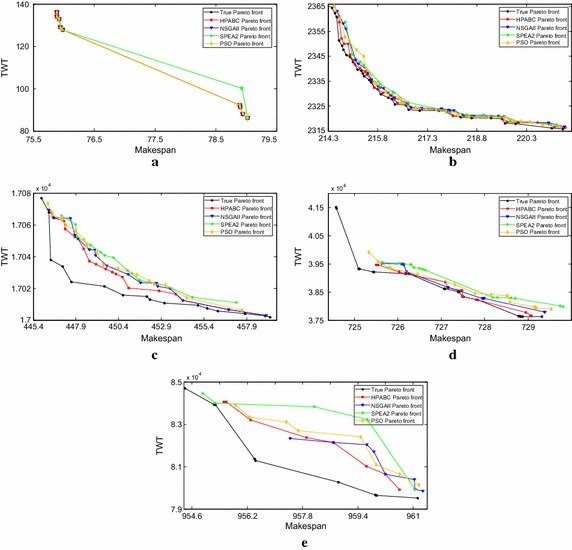
Pareto front of HPABC, SPEA2, PSO and NSGAII algorithm for different size of problems. **a** Pareto front of HPABC, SPEA2, PSO and NSGAII algorithm for the 2 groups case problem of small size problems. **b** Pareto front of HPABC, SPEA2, PSO and NSGAII algorithm for the 5 groups case problem of small size problems. **c** Pareto front of HPABC, SPEA2, PSO and NSGAII algorithm for the 10 groups case problem of small size problems. **d** Pareto front of HPABC, SPEA2, PSO and NSGAII algorithm for the 15 groups case problem of small size problems. **e** Pareto front of HPABC, SPEA2, PSO and NSGAII algorithm for the 19 groups case problem of small size problems

The performance of proposed HPABC, SPEA2, PSO and NSGA II algorithm on the basis of their Pareto fronts for an instance from different categories of problems are illustrated in Fig. 12. It can be seen from these figures that in different size of problems, the Pareto fronts generated by the proposed HPABC algorithm are always nearer to the true Pareto front which turns out HPABC is better than SPEA2, PSO and NSGAII for the proposed problem in current research.

Pareto fronts of HPABC, SPEA2, PSO and NSGAII algorithms for one of the small size problems, small medium size problems, medium size problems, large medium and large size problems are indicated in Fig. 12a–e respectively. It is shown in Fig. 12a that, Pareto fronts obtained from HPABC, PSO and NSGAII coincide because they can get all the true Pareto solutions for the instance with 2 groups of small size problems. While SPEA2 may only find some of the true Pareto solutions. It can be seen from Fig. 12b that for the 5 groups instance of small medium size problems, most Pareto points found by HPABC are nearer to the true Pareto front. From Fig. 12c it can be seen that the Pareto front obtained from HPABC for10 groups instance of medium size is much nearer to the true Pareto front as compared to the fronts obtained from NSGA II, PSO and SPEA 2 algorithms for the same problem. Figure 12d indicates that the solution points of HPABC are very near to the true Pareto front for the case with 15 groups of large medium size while SPEA2, PSO and NSGAII are a little bit far with respect to HPABC. Meanwhile, HPABC can obtain more number of Pareto solutions for the current instance of large medium size as compared to SPEA2, PSO and NSGAII. Furthermore, it can be seen from Fig. 12e for lager size problem with 19 groups, the Pareto points of HPABC can dominate much more Pareto points of SPEA2, PSO and NSGAII. However, SPEA2, PSO and NSGAII may randomly obtain a few point better than HPABC. These results indicate that their results might not be stable to find the optimal solutions for large size problems consistently. In conclusion, all Pareto results obtained from HPABC outperforms SPEA2, PSO and NSGAII and can generate optimal Pareto front for different category of problems in current study.

## Conclusions

Group scheduling problem has got lots of attentions in recent years because it is significant for efficient and cost effective production environment. In current study a single machine group scheduling problem involving SDS time and learning effect, is proposed here. Furthermore, multi objective optimization is considered to minimize the makespan and the total weighted tardiness time simultaneously due to the desire of multiple conflicting objectives at the same time in real environment. Moreover, a hybrid Pareto artificial bee colony (HPABC) algorithm, which integrates the original ABC algorithm with some steps of genetic algorithm and the Pareto optimality, is presented to get Pareto solution of the multiple objectives.

The effective parameters of the proposed HPABC algorithm are tuned with robust experimental design procedure using Taguchi method. In this method five different sizes (small, small medium, medium, large medium and large) of test problems involving 19 instances are presented for the current problem. The proposed HPABC algorithm parameters are identified and tuned for each size of problems with Taguchi method. In order to assess the performance of HPABC algorithm, the computational experiments are carried out and the results based on diversity and quality measures, GD value, SP value and Pareto front reveal that the proposed HPABC outperforms SPEA2, PSO and NSGAII comprehensively. Future research can be extended by taking into account of simultaneous sequence dependent group scheduling and lot-sizing scheduling together. In addition, more practical applications need to be considered, e.g., multi-parallel machine scheduling, the uncertain arrival time of jobs and the machine reliability, etc. Furthermore, the proposed HPABC algorithm is desired to be further developed in terms of convergence and diversity.
